# Supplementary material for: Pre-Treatment, Extraction Solvent, and Color Stability of Anthocyanins from Purple Sweetpotato
Source: Foods. 2024 Mar 8;13(6):833. doi: 10.3390/foods13060833 (PMC10969496; doi:10.3390/foods13060833)
Supplement: Supplementary file 1 [file foods-13-00833-s001.zip › foods-2875742-supplementary.pdf]

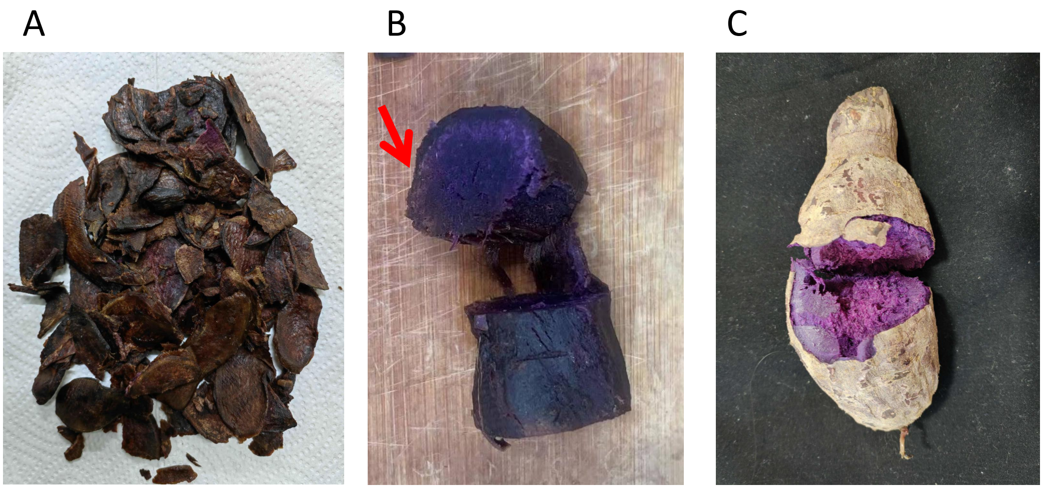

Figure S1. Appearance of the purple sweetpotato by different pre-treatments after steaming.
(A) peeled and sliced for SD-s, (B) peeled for SD-p, and (C) whole without peeling for SD-w. The red arrow showed the brown edge for the peeled sweetpotato sample.

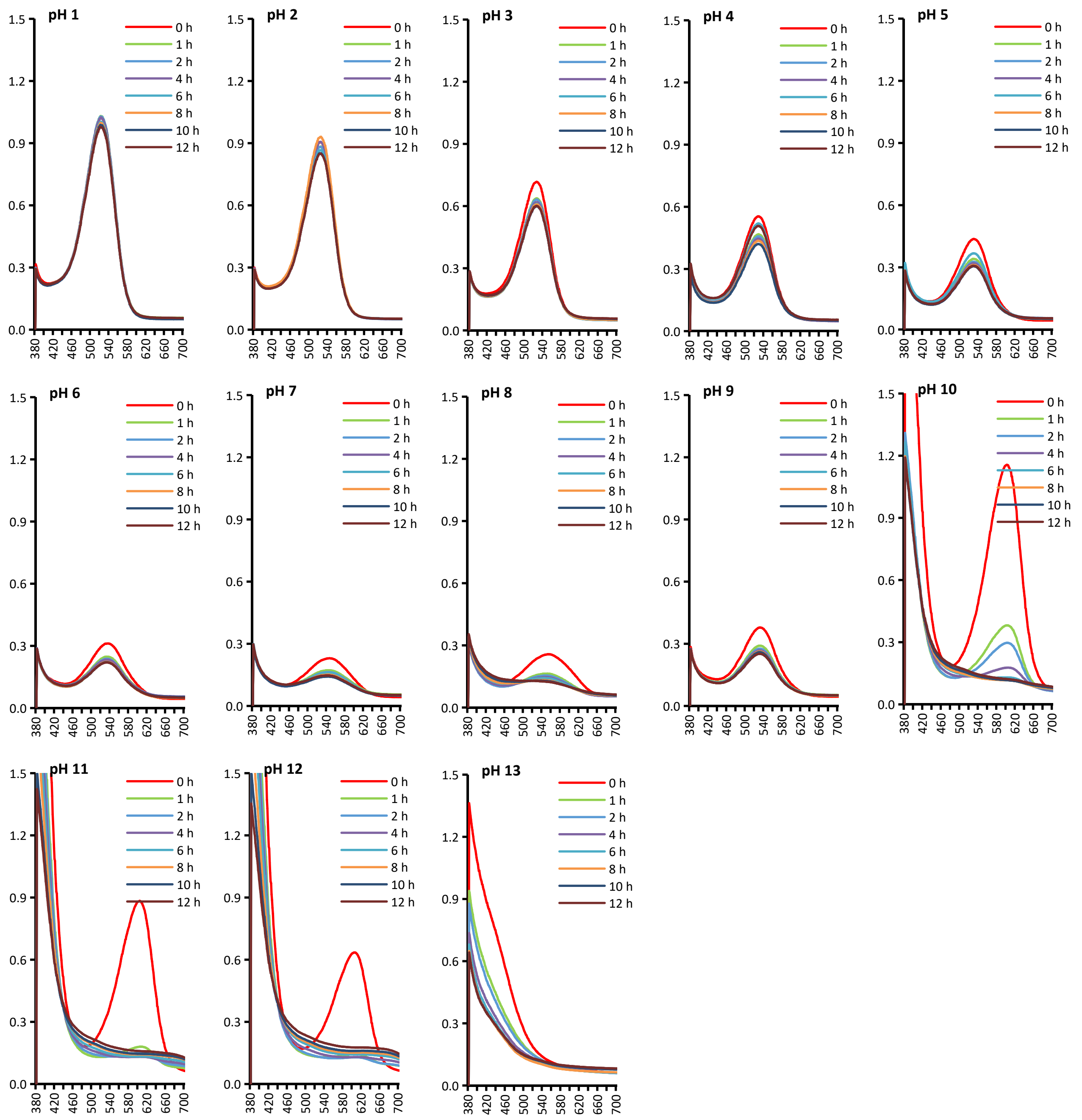

Figure S2. Spectra for PSPA degradation in solutions at different pHs at 65℃ within 12 hours.

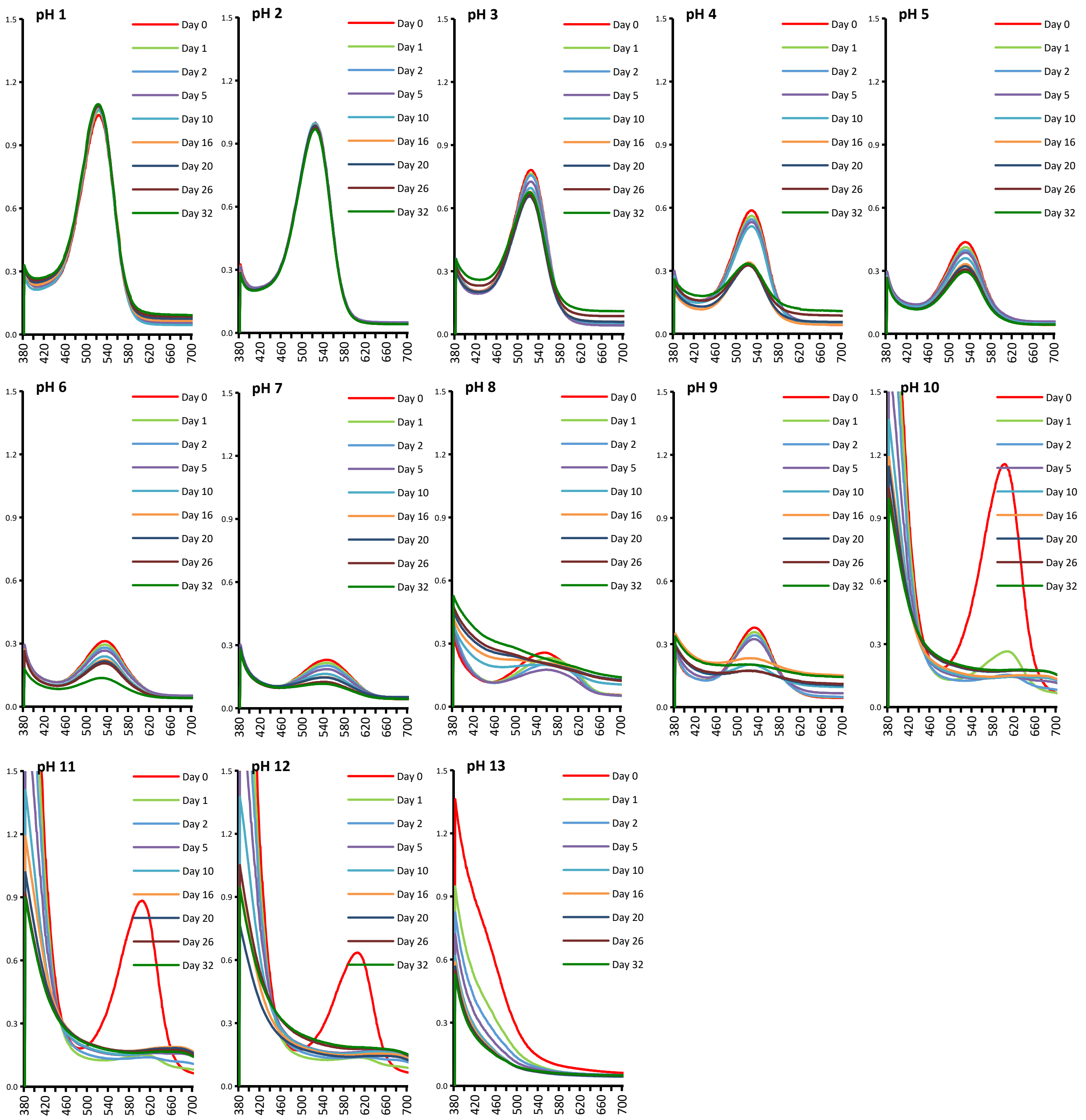

Figure S3. Spectra for PSPA degradation in solutions at different pHs at 25℃ within 32 days.
